# Genome-wide linkage analysis of inguinal hernia in pigs using affected sib pairs

**DOI:** 10.1186/1471-2156-7-25

**Published:** 2006-05-03

**Authors:** Eli Grindflek, Maren Moe, Helge Taubert, Henner Simianer, Sigbjørn Lien, Thomas Moen

**Affiliations:** 1 The Norwegian Pig Breeders Association (NORSVIN), Hamar, Norway; 2 Centre for Integrative Genetics, Norwegian University of Life Sciences, Aas, Norway; 3 Department of Animal and Aquacultural Sciences, Norwegian University of Life Sciences, Aas, Norway; 4 Institute of Animal Breeding and Genetics, Georg-August University of Goettingen, Goettingen, Germany; 5 AKVAFORSK, Aas, Norway

## Abstract

**Background:**

Inguinal and scrotal hernias are of great concern to pig producers, and lead to poor animal welfare and severe economic loss. Selection against these conditions is highly preferable, but at this time no gene, Quantitative Trait Loci (QTL), or mode of inheritance has been identified in pigs or in any other species. Therefore, a complete genome scan was performed in order to identify genomic regions affecting inguinal and scrotal hernias in pigs. Records from seedstock breeding farms were collected. No clinical examinations were executed on the pigs and there was therefore no distinction between inguinal and scrotal hernias. The genome scan utilised affected sib pairs (ASP), and the data was analysed using both an ASP test based on Non-parametric Linkage (NPL) analysis, and a Transmission Disequilibrium Test (TDT).

**Results:**

Significant QTLs (p < 0.01) were detected on 8 out of 19 porcine chromosomes. The most promising QTLs, however, were detected in SSC1, SSC2, SSC5, SSC6, SSC15, SSC17 and SSCX; all of these regions showed either statistical significance with both statistical methods, or convincing significance with one of the methods. Haplotypes from these suggestive QTL regions were constructed and analysed with TDT. Of these, six different haplotypes were found to be differently transmitted (p < 0.01) to healthy and affected pigs. The most interesting result was one haplotype on SSC5 that was found to be transmitted to hernia pigs with four times higher frequency than to healthy pigs (p < 0.00005).

**Conclusion:**

For the first time in any species, a genome scan has revealed suggestive QTLs for inguinal and scrotal hernias. While this study permitted the detection of chromosomal regions only, it is interesting to note that several promising candidate genes, including INSL3, MIS, and CGRP, are located within the highly significant QTL regions. Further studies are required in order to narrow down the suggestive QTL regions, investigate the candidate genes, and to confirm the suggestive QTLs in other populations. The haplotype associated with inguinal and scrotal hernias may help in achieving selection against the disorder.

## Background

The occurrence of hernias is a significant problem facing pig producers, and leads to poor animal welfare and severe economic loss. The most commonly occurring hernias are *hernia inguinalis *and *hernia scrotalis *which occur within the pig population at frequencies from 1.7% to 6.7% [1]. By definition, an inguinal hernia describes a situation where hernial contents are present in the inguinal canal, whereas scrotal hernia refers to a situation where hernial contents are present in the scrotum. In these conditions, most frequently the distal jejunum and ileum pass through the vaginal ring and enter the inguinal canal. Small colon and omentum can also herniate but this occurs less frequently. Inguinal and scrotal hernias are further subdivided, in human medicine, as indirect or direct. An indirect hernia refers to the passage of intestinal loops through the vaginal ring into the vaginal tunic. While a direct hernia refers to the passage of intestinal loops through a fascial defect near the vaginal ring, resulting in a situation where intestinal loops pass through the inguinal canal but are not covered by the vaginal tunic. The inguinal canal is a short passage running through the inferior part of the interior abdominal wall. Under normal conditions its function is to allow the testes, which develop in the lumbar region of the abdomen, to migrate to the scrotum. The testes descend from the anterior abdominal wall through the *processus vaginalis *via a propulsive force generated by muscles derived from the gubernaculums, a ligament linking the testes and scrotum. Development of inguinal and scrotal hernias usually results from failed obliteration of the *processus vaginalis *after descent of the testis [2], or from failed involution at the internal inguinal ring [3]. Complication may also occur, in the form of intestinal obstruction or strangulation [4]. Furthermore, in many cases the undescended testes are associated with a patent *processus vaginalis *because the *processus vaginalis *does not obliterate unless the testis reach the scrotum [5]. This particular form of inguinal and scrotal hernias is correlated with the occurrence of cryptorchism [1], and is a concern for the pig breeding industry as well.

Several studies have shown that genetic factors are involved in the development of inguinal and scrotal hernias [6,7], however the mode of inheritance has not yet been clarified. Estimated h^2 ^for these types of hernia range from 0.20 [8] to 0.86 [9]. Several studies have investigated genes involved in the control of testicular descent, obliteration of *processus vaginalis *and in the closing of the inguinal ring; by association, these genes may also be involved in the occurrence of inguinal and scrotal hernias. Specifically, Insulin-like receptor 3 (INSL3), Müllerian inhibiting substance (MIS), and relaxin are all involved in gubernacular growth [10], while the calcitonin gene-related peptide (CGRP) released from the genitofemoral nerve, may be responsible for failed fusion and disappearance of *processus vaginalis *[11,12].

The aim of this study was to identify genomic regions affecting the frequency of inguinal and scrotal hernias in pigs. Records from seedstock breeding farms were used in this study. No clinical examinations were executed on the pigs and therefore, there was no distinction between inguinal and scrotal hernias. Examination of these regions may reveal causative genes responsible for the trait, which would be valuable information for use in Marker-Assisted Selection (MAS) schemes directed at decreasing hernia prevalence. Previously, parametric linkage analysis applied to large pedigrees containing many affected individuals has helped in the identification of genes with high penetrance [13]. However, for those diseases lacking a clear Mendelian inheritance pattern, or caused by several genes of low to moderate penetrance, nonparametric analysis can be a more robust and successful alternative. Crucially, linkage analysis requires correct assumptions to be made regarding the inheritance model and allele frequencies of the susceptibility alleles, and incorrect model specification will result in a loss of power and a bias in the estimation of recombination fraction [14]. Nonparametric methods, by comparison, require no such assumptions to be made, and we consider this to be better suited approach for our analysis of small sets of affected relatives. The Transmission/Disequilibrium Test (TDT) [15] was used to investigate whether the identified hernia-linked markers were associated with the trait on a population level. In the present article, we describe the results of the first published genome-wide scan of inguinal and scrotal hernias in pigs, as well as one of the first genome scans in livestock populations to use the affected sib-pair design.

## Results

### Linkage analysis

**Figure 1:**
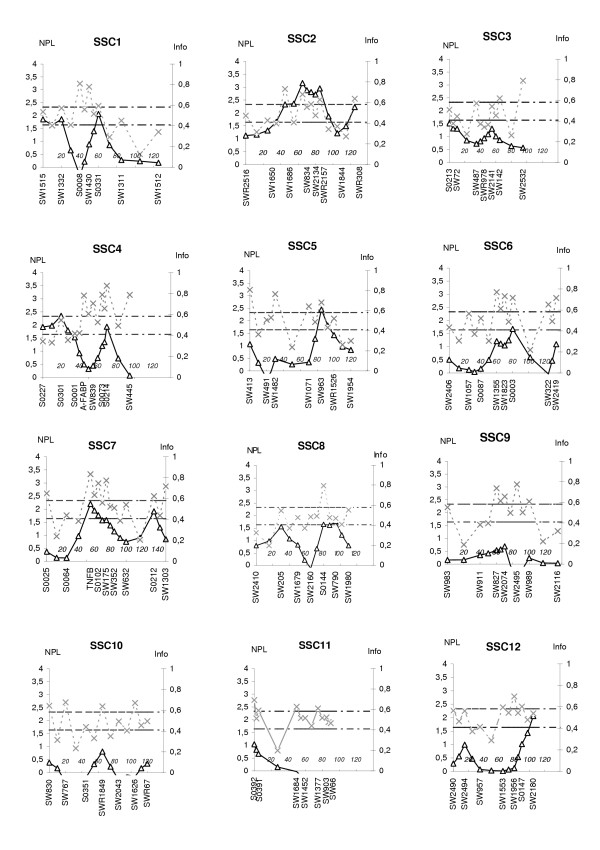
**Linkage results of the total genome scan for inguinal and scrotal hernias, showing sus scrofa chromosomes (SSC) 1 to 12**. The bold line shows multipoint NPL score (scale on left) while the dotted line shows the information content (scale on right) for each chromosome. The threshold values P = 0.05 and P = 0.01 are shown as horizontal lines at NPL = 1.64 and NPL = 2.33, respectively. Marker positions and names are shown on the X axis.

**Figure 2:**
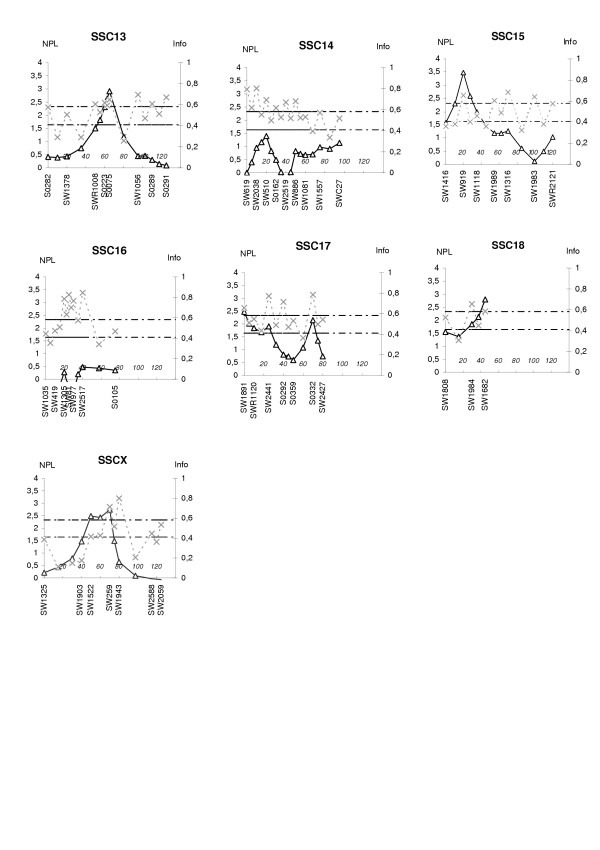
**Linkage results of the total genome scan for inguinal and scrotal hernias, showing sus scrofa chromosomes (SSC) 13 to X**. The bold line shows multipoint NPL score (scale on left) while the dotted line shows the information content (scale on right) for each chromosome. The threshold values P = 0.05 and P = 0.01 are shown as horizontal lines at NPL = 1.64 and NPL = 2.33, respectively. Marker positions and names are shown on the X axis.

The linkage analysis was performed using an affected sib pair (ASP) test. Figure 1 and 2 shows the profiles of the multipoint NPL scores and the respective information content (Info) for each chromosome. The average information content was 0.52, which is a similar value to those found in previous studies in swine [16] and humans [17]. Two chromosomal regions had linkage score exceeding the nominal significance level of p < 0.001. The most significant regions were found on SSC2, with a multipoint NPL score of 3.16 at marker SW834, and on SSC15 which showed an NPL score of 3.47 at marker SW919. Four markers on SSC2 reached a significance level of p < 0.01 (NPL score larger than 2.33); these four markers spanned a chromosomal region of 40 cM. In addition, the linkage score exceeded the nominal significance level of p < 0.01 for six chromosomes, with highest maximum multipoint NPL scores of 2.35 at marker S0301 on SSC4, 2.45 at marker SW963 on SSC5, 2.92 at marker S0075 on SSC13, 2.47 at marker SW1891 on SSC17, 2.80 at marker SW1808 on SSC18, and 2.74 at marker SW259 on SSCX. Finally, the nominal significance level of p < 0.05 was reached on four chromosomal regions with highest maximum multipoint NPL scores of 2.06 at marker S0331 on SSC1, 1.68 at marker S0003 on SSC6, 2.20 at marker TNFB on SSC7, and 2.06 at marker SW2180 on SSC12. Results with significance level above 5% are presented in Table 1.

**Table 1:** Comparison of results of affected sib pair test (ASP) and transmission disequilibrium test (TDT). The chromosome positions and single-point levels of significance are shown for all significant markers in the genome scan. The significant p-values (1%) are in bold.

**Chromosome**	**Marker**	**Position (cM)**	**P-value, ASP**	**P-value, TDT**
SSC1	SW1515	0	0.05	0.07
	SW1332	20	0.05	**0.005**
	S0331	60	0.05	0.05
SSC2	SW1686	45	**0.01**	0.23
	SW834	65	**0.001**	0.86
	SW2134	75	**0.002**	0.17
	SWR2157	85	**0.001**	0.90
	SWR308	125	**0.01**	0.06
SSC4	S0227	0	0.05	0.49
	S0301	20	**0.01**	0.53
	S0214	70	0.05	0.61
SSC5	SW963 *	80	**0.01**	**0.005**
SSC6	SW1355 *	60	0.11	**0.0002**
	S0003	80	0.05	0.18
SSC7	TNFB	55	0.05	0.10
	S0212	135	0.05	0.05
SSC12	SW2180	105	0.05	0.18
SSC13	S0223	60	**0.01**	0.40
	S0075	65	**0.01**	0.93
SSC15	SW919	20	**0.001**	0.02
	SW1118	35	0.05	**0.01**
SSC17	SW1891	0	**0.01**	**0.01**
	SWR1120	10	0.05	0.74
	SW2441	25	0.05	0.11
	S0332	70	0.05	**0.01**
SSC18	SW1984	30	0.05	0.17
	SW1682	45	**0.01**	0.72
SSCX	SW1522	50	**0.006**	0.28
	SW259	70	**0.003**	0.53

* Markers with chromosomewise significance of p < 0.01 from the TDT analyses

### TDT analysis

TDT analysis was applied to all loci in the data set. The significant loci are presented in Table 1. At the TDT-significant loci, TDT was also performed on the allele level, to identify individual hernia-associated alleles (Table 2). Markers displaying significance at a nominal value of p < 0.05 were found on SSC1, SSC5, SSC6, SSC7, SSC15, and SSC17. A chromosome-wide significance of p < 0.01 was found for the two markers; SW963 on SSC5 and SW1355 on SSC6. All TDT-significant markers were also significant in the ASP test, with the exception of SW1355 on chromosome 6, which was also the most significant marker found in the TDT.

**Table 2:** Counts used as input for TDT, with results from single-allele TDT. The counts are the number of times that an allele was transmitted/not transmitted from a heterozygous parent carrying that allele to a hernia affected/not affected offspring. Nominal significance levels were used.

**Chromosome**	**Marker**	**Allele**	**Affected**		**Resistant**		**TDT**	**P-value**
					
			Transmit.	Not transmit.	Transmit.	Not transmit.		
SSC1	S0331	1	49	14	27	20	7.13	**0.007**
		2	10	25	11	13	2.86	0.09
		3	13	25	10	15	0.78	0.38
		4	0	8	2	2	5.33	**0.02**
		5	4	4	3	3	0.00	1.00
	SW1332	1	22	35	7	23	0.10	0.75
		2	54	24	25	16	3.71	**0.05**
		3	1	14	5	2	11.64	**0.0006**
		4	1	5	4	0	6.40	**0.011**
SSC5	SW963	1	53	25	13	25	13.79	**0.0002**
		2	8	10	3	7	0.14	0.70
		3	27	37	17	10	3.18	0.07
		4	27	35	19	14	1.78	0.18
		5	8	10	7	1	2.46	0.12
		6	1	5	1	3	0.40	0.53
		7	0	2	1	1	1.00	0.32
SSC6	SW1355	1	48	70	48	19	14.06	**0.0002**
		2	16	14	1	17	6.75	**0.009**
		3	41	35	18	21	0.70	0.40
		4	38	24	12	22	6.00	**0.01**
SSC7	S0212	1	9	11	8	7	0.26	0.61
		2	52	29	17	20	5.73	**0.01**
		3	1	12	0	7	0.80	0.37
		4	18	21	15	7	1.98	0.16
		5	25	33	18	12	2.23	0.14
		7	5	4	1	6	2.25	0.13
SSC15	SW1118	1	40	21	15	24	7.84	**0.005**
		2	11	16	8	7	0.86	0.35
		3	9	17	10	6	3.43	0.06
		4	2	9	3	3	2.88	0.09
		5	7	6	6	2	0.43	0.51
	SW919	1	52	35	13	27	7.57	**0.006**
		2	18	33	15	18	1.71	0.19
		3	37	35	32	7	4.77	0.03
		4	32	34	18	16	0.16	0.69
		5	8	10	0	10	2.29	0.13
		6	1	1	1	1	0.00	1.00
SSC17	S0332	1	24	31	14	12	1.00	0.32
		2	1	21	0	10	3.13	0.08
		4	30	42	15	34	0.40	0.52
		5	7	20	9	4	8.10	**0.004**
	SW1891	1	60	39	32	29	2.03	0.15
		2	27	41	28	7	11.89	**0.0005**
		3	19	24	9	20	0.50	0.48
		4	25	27	9	22	1.46	0.22

The loci showing significance for both ASP and TDT, and the regions showing high significance (p < 0.005) with only ASP or TDT, were considered to be the most interesting for further analyses. Haplotypes from these regions were constructed and TDT analysis was used to identify those haplotypes most strongly associated with hernia. Six different haplotypes were found to be differently transmitted (p < 0.01). One haplotype on SSC5 was transmitted four times more frequently to pigs with hernias than to healthy pigs (p < 0.00005). Other haplotypes, showing an increased frequency of transmission to affected pigs (p < 0.01) were found on SSC6, SSC15 and SSC17. Table 3 shows the markers included in the haplotypes, the number of affected/non-affected animals with the transmitted/non-transmitted haplotype, and the p-values. Distributions belonging only to the significant haplotypes are shown.

**Table 3:** The significant haplotypes from the most significant QTL regions were analysed by TDT. The counts are the number of times that an allele was transmitted/not transmitted from a heterozygous parent carrying that allele to a hernia affected/not affected offspring. Nominal significance levels were used.

**Chromosome**	**Markers in the haplotype**	**Affected**		**Non-affected**		**TDT**	**P-value**
				
		Transmit.	Not transmit.	Transmit.	Not transmit.		
SSC5	SW963-SWR1526	41	16	10	24	16.7	0.00004
SSC6	SW1355-SW1823-S0003	30	13	6	13	9.3	0.002
SSC15	SW919-SW1118	56	36	16	28	7.5	0.006
		5	15	8	3	7.3	0.007
SSC17 _ 1	SW1891-SWR1120	5	15	8	3	7.3	0.007
SSC17 _ 2	S0359-S0332	58	26	21	22	8.6	0.003

## Discussion

Our genomewide scan for QTLs affecting incidence of hernia revealed 9 genomic regions on 8 chromosomes using ASP test (p < 0.01), and 6 genomic regions on 6 chromosomes using TDT (p < 0.01). Two of these regions, on SSC5 and SSC17, were significant (p < 0.01) when analysed by both statistical methods (Table 1). Five regions located on SSC1, SSC2, SSC6, SSC15 and SSCX were found to be highly significant (p < 0.005) from either the ASP test or the TDT. We consider these seven regions to be those most likely involved in genetic susceptibility to hernia. Two different statistical methods were used (ASP and TDT) in order to take advantage of their complementary qualities in detecting genomic regions affecting defect/disease traits. The ASP method tests for markers linked to genes affecting deformity status, while the TDT method tests for markers that are both linked to these genes and associated with a particular trait on a population level. Essentially, the TDT is a fine-mapping method requiring linkage disequilibrium (LD) between causative polymorphisms and the nearest markers. In this study however, the TDT has been used on families with more than one (affected or unaffected) offspring per parental pair; under such conditions the TDT is, to some extent, a test for linkage, although the power of the test still increases greatly with the amount of LD [18]. Therefore, for our application, the ASP and TDT are seen as two different tests for genes linked to the trait, possessing complementary strengths and weaknesses. In particular, the ASP is expected to be the more powerful test when the causative polymorphism is linked to, but not in LD with, the nearest marker, whereas the TDT would have greater power if some amount of LD were present. Other factors, such as penetrance and allele frequencies in causative polymorphisms and markers, may also lead to differences in power between the two tests [19]. Previous studies have shown that the two methods may give different results. For example, the TDT detected a very strong association between *diabetes mellitus *and a marker in the 5' region of the insulin gene [20,21], although this marker had earlier been shown to have no association in an ASP-based test for linkage [22,23]. In our study several genomic regions are highly significant for the ASP test but not for TDT (Table 1). As the ASP test can detect linkage over long distances, these results may be explained by markers being linked to, but not in close LD with, the causative polymorphisms. The negative TDT results should therefore not be interpreted as a negation of the findings from the ASP test, but rather as indications that the markers are not located in sufficient proximity to the causative polymorphisms. In fact, with the marker densities used in this study, ASP-positive but TDT-negative markers are expected. However, one marker that was strongly positive for the TDT test (SW1355 on SSC6, p < 0.0002), was negative for the ASP test. This may be explained by the fact that in contrast to the ASP, the TDT method can detect association with high power when homozygotes for a susceptibility allele have as little as 2 to 4-fold greater disease risk than homozygotes for the normal or wild-type allele, while parameters such as penetrance and allele frequencies may also favour the TDT rather than the ASP test [19].

The causative mutations we are seeking do not occur in isolation, but with a number of different mutations within the region. Linkage disequilibrium (LD) deteriorates over successive generation because of recombination, but the mutation will remain in LD with closely linked markers. It is therefore, possible to define a risk modifying haplotype in a region of the genome surrounding the disease mutation. Once multiple single-point associations within a region have been identified, haplotype based tests for disease associations may be performed. In this investigation, haplotypes consisting of marker alleles in the putative QTL regions were investigated using TDT analysis. Several haplotypes occurred with a higher frequency in the affected pigs compared to the unaffected pigs, indicating that these haplotypes are associated with the occurrence of inguinal and/or scrotal hernias in pigs.

While most porcine genes are still not mapped, the determination of homologous synteny blocks between human and porcine chromosomes allows us to propose some candidate genes for inguinal and scrotal hernias using comparative genomics [24-27]. The comparative regions and possible candidate genes within these regions are shown in Table 4. As mentioned previously, inguinal and scrotal hernias may arise from a failure to achieve complete closure of the *processus vaginalis*, and a failure of involution at the internal inguinal ring [3]. In addition, the genitofemoral nerve, the caudal ligament gubernaculum, and controllers of testicular descent may control subsequent closure of the *processus vaginalis*. Finally, regulation of collagen metabolism has been shown to play an important role in the development of inguinal and scrotal hernias [28,29]. Using this information as a guide it is possible to anticipate that certain candidate genes may be located in some of the putative QTL regions. Three interesting candidate genes (INSL3, CGRP, MIS) have been mapped to homologous regions in the putative QTL on SSC2 [30-32]. From studies using transgenic knockout mice [33] and humans [34], INSL3 is believed to be the primary testicular hormone inducing gubernacular development, and has been associated with occurrences of inguinal hernia in female mice [7]. No significant associations were, however, observed between two polymorphisms in the INSL3 gene and inguinal hernia in pigs [35]. The CGRP is active in the induction of *processus vaginalis *fusion, a process involving substantial tissue remodelling and the characteristic transformation of *processus vaginalis *epithelium [6,12]. Finally, MIS has been shown to be involved in the swelling reaction of gubernaculum occurring during the first phase of testicular descent [12,36]. Moreover, MIS together with testosterone and INSL3, controls sex differentiation-like development of internal and external reproductive organs and the acquisition of male secondary sex characteristics [37]. Additional candidate genes were found in putative QTL regions using the network browser option in PubGene [38]. Estrogen receptor 1 (ESR1) and collagen typeIXα (COL9A1) are both candidate genes located to human (HAS) 6q25 region, which is comparative to the putative QTL on SSC1. Coveney et al. [39] demonstrated that estrogen has profound effects on development of the internal genitalia in male marsupials, preventing inguinal closure and interfering with testicular descent, and ESR1 knockout mice lack gubernaculum development in late stages of maturity [40]. Pathological changes in collagen are also involved in development of a hernia [41]. It is therefore interesting to notice that in cartilage, type IX collagen (COL9A1) molecules are covalently crosslinked to type II collagen molecules, which represents approximately 85% of the collagen contained in hyaline cartilage 3 [42]. Interestingly, the gene (COL2A1) coding for collagen type II has been mapped to a region in HSA12 which is comparative to the putative QTL region on SSC5. Candidate genes were also seen in the QTL regions on SSC6 (INSL5) and SSC7 (CYP19A1). No obvious candidate genes were seen in the genomic regions of SSC7q24-26, SSC15 and SSC17 or in the comparative genomic regions in human. The current comparative maps, however, do not allow precise prediction of the likely map position.

**Table 4:** Putative candidate genes in candidate QTL regions for inguinal and scrotal hernias. The most promising regions on SSC1, SSC2, SSC5, SSC6, SSC7, SSC15 and SSC17 are shown, as well as the homologous regions in human.

**QTL region**^1^	**Homologous regions in human**^2^	**Putative candidate genes**
SSC1 p25 – q14	HSA6 q25	Collagen typeIXα (COL9A1), Estrogen receptor 1 (ESR1)
	HSA18 p11.2 – p11.32	
SSC2 p14 – q23	HSA5 q12 – q35	
	HSA11 q13 – p15	Calcitonin gene-related peptide (CGRP)
	HSA19 p13.1 – p13.3	Insulin like hormone 3 (INSL3), Mullerian-inhibiting substance (MIS)
SSC5 q11 – q22	HSA12 q11.2 – p13	Collagen type II (COL2A1)
SSC6 q27 – q32	HSA1 p22 – p36.3	Insulin-like hormone 5 (INSL5)
	HSA18 q12 – p11.32	
SSC7 q11 – q15	HSA15 q12 – q26	Cytochrome P450, family19A1 (CYP19A1)
SSC7 q24 – q26	HSA14 q11.2 – q32	
SSC15	HSA2 q11.2 – q21	
SSC17 p11 and p21	HSA5 and HSA20	

^1^Based on the cytogenetic maps we suggest where the microsatellite markers are physically mapped to the porcine genome [48,49]

^2^This is seen from comparative mapping between pig and human [24,25,26,27,50]

To our knowledge, this is the first report of a whole genome scan for markers associated with inguinal and scrotal hernias, and one of the first genetic studies using ASP in livestock. The results reported in this study will encourage us to perform follow-up studies using finemapping approaches in an extended genetic material. Our goal is to discover the causal mutations underlying inguinal and/or scrotal hernias, and to look for shared ancestral haplotypes among pigs with these types of hernias. Ultimately we wish to understand the epistatic interactions between inguinal/scrotal hernia genes, and the interactions between genes and their environment, and their impact on a variety of relevant cofactors associated with this defect. We believe the data contained in the present study represents an important and useful basis to further search for candidate genes causing inguinal and scrotal hernias in pigs. The outcome from these inquiries may also be of relevance to human hernia conditions.

## Conclusion

For the first time in any species, a genome scan has revealed suggestive QTLs for inguinal and scrotal hernias. Significant QTLs were detected in 8 out of 19 porcine chromosomes (p < 0.01). However, the most promising QTLs were found on SSC1, SSC2, SSC5, SSC6, SSC15, SSC17 and SSCX. These regions were found to be significant when using both statistical methods (ASP and TDT), or with a convincing high significance for either of the methods. Haplotypes from the suggestive QTL regions were constructed and used in TDT analysis. Six different haplotypes were found to be differently transmitted when contrasting hernia pigs with healthy pigs (p < 0.01). One haplotype on SSC5 was transmitted four times more frequently to hernia pigs than to healthy pigs (p < 0.00005). Only chromosomal regions could be detected in this study, but it is noteworthy that several promising candidate genes, including INSL3, MIS, and CGRP, are all located in a highly significant QTL region. Further studies must be performed to narrow down the suggestive QTL regions, investigate the candidate genes, and finally to confirm the suggestive QTLs in other populations.

## Methods

### Animals and phenotypic records

Due to a generally low incidence of *hernia inguinalis *or *hernia scrotalis*, it was necessary to utilise a collection system involving all the breeding farms in Norsvin (Hamar, Norway) in order to access a sufficient number of affected sib pairs (ASP). The power calculations and minimum number of affected sib pairs required for this study was evaluated based on a method suggested by Simianer and Stricker [43]. Blood samples were collected from a total of 100 full- and half-sib Norwegian Landrace piglets displaying inguinal and/or scrotal hernia, along with blood from 51 phenotypically unaffected full- and half-sibs. To supplement these samples, DNA from 27 fullsib Danish Landrace piglets affected with inguinal and/or scrotal hernias, and 16 unaffected siblings, was provided from Danish Slaughterhouses (Denmark). In total there were 194 samples, distributed on 52 litters containing 103 affected sib pairs, with each litter containing 2 to 5 affected piglets. Two affected siblings from one litters gives 1 ASP, three affected siblings from one litters gives 3 ASP, and so on. In addition to affected animals and 1 or 2 unaffected siblings, we obtained blood specimens from all available parents (31 sires and 46 dams). In several cases these were parent to more than one affected litter. Samples were obtained from 35 pig breeding farms altogether, and several sires were used on different farms.

When a farmer reported an affected animal, diagnostic procedures were performed by a breeding consultant from Norsvin. Without clinical examination, it is impossible to distinguish direct hernia from indirect hernia, or inguinal hernia from scrotal hernia. No clinical examinations were executed, so the diagnosis of hernia includes both inguinal and scrotal hernias, as well as both indirect and direct hernias. In human and horses, inguinal and scrotal hernias are almost exclusively of the indirect type [44], therefore we expect most of the hernias to be of the indirect type in pigs also.

### Genotyping

Genomic DNA was extracted from a total of 282 pigs (parents and offspring). 130 microsatellite markers from the autosomal chromosomes and 7 microsatellites from the X/Y chromosome were selected from the USMARC Genome Database (2000), based on position, ease of scoring and number of alleles [45]. The markers were amplified using PCR; the reaction volume was 10 μl, containing 50 ng swine genomic DNA template, 10 × PCR buffer, 15 mM MgCl_2_, 2 mM dNTP, 0.01 μm of each primer and 5U Gold Taq polymerase (Applied Biosystems, Foster City, CA, USA). The PCR program included an initial denaturation step of 10 sec at 95°C, followed by 40 cycles of 30 sec at 94°C, and 30 sec at 55°C-62°C, with a final extension step of 5 min at 73°C. Fragment lengths were determined either by 1) electrophoresing PCR product through a 6% denaturating polyacrylamide gel in an ABI-377 DNA sequencer (ABI, Perkin Elmer, Foster City, CA, USA), where each forward primer was 5'-labeled with one of three fluorophores (TET, HEX or 6-FAM), or 2) with capillary electrophoresis on an ABI 3730 DNA sequencer (ABI, Perkin Elmer, Foster City, CA, USA), each forward primer 5'-labeled with one of four fluorophores (NED, PET, VIC or 6-FAM). Genescan and Genotyper version 3.7 software or GeneMapper 3.0 (ABI, Perkin Elmer, Foster City, CA, USA) was used for genotype scoring. A computer program was written in house to control for Mendelian inconsistencies in the marker data.

### Statistical analysis

#### Marker map

The family material used in the study was not suitable for constructing genetic maps. Therefore the order of the 137 microsatellites chosen for the genome scan, and the genetic distances between them, were taken from the USMARC Genome Database (2000). The markers were evenly distributed across the 18 autosomal chromosomes and the X-chromosome, including one marker in the pseudoautosomal region. The total length of the map spanned by the marker set was 2335 cM. Average marker heterozygosity was 0.57 and the mean intermarker spacing was 17 cM. The locations of markers are shown in Figure 1 and 2.

#### Allele frequencies

A non-parametric linkage analysis using the software ALLEGRO 1.0 [46], requires allele frequencies for markers if one parent is unknown and the transmission of alleles are not unambiguously traceable from the marker genotypes of the other parent and offspring. In our animal material some of the dams and a few sires were missing. Marker allele frequencies in the population were estimated from our data set using a computer program made for this purpose.

#### Linkage analysis

Two different test statistics were applied for linkage analysis using the software package ALLEGRO 1.0 [46]. The first statistic was numbers of shared alleles (NSA), i.e. the number of times that two affected full-sibs displayed identical alleles at the two flanking markers. Under the null hypothesis of no correlation between marker genotypes and defect status, two affected full-sibs would be expected to share half their alleles. Under the alternative hypothesis of a linked gene affecting defect prevalence, the proportion of alleles being identical by descent (and accordingly, identical by state) would be expected to be higher. The second test statistic considers how many of the shared alleles of an affected sib-pair are identical by the descent (IBD). Multipoint linkage analysis of the genome scan data was performed with the non-parametric affected relative method of ALLEGRO 1.0 [46], which also provides multipoint NPL scores and the corresponding p-values [47].

#### Transmission/Disequilibrium test

Transmission/Disequilibrium Tests [21] were carried out according to Lazzeroni and Lange [18]. At every locus, the TDT statistic was calculated as

T=∑j=1ltja+cju−(tju+cja)tja+cju+tju+cja     [Eq.1]
 MathType@MTEF@5@5@+=feaafiart1ev1aaatCvAUfKttLearuWrP9MDH5MBPbIqV92AaeXatLxBI9gBaebbnrfifHhDYfgasaacH8akY=wiFfYdH8Gipec8Eeeu0xXdbba9frFj0=OqFfea0dXdd9vqai=hGuQ8kuc9pgc9s8qqaq=dirpe0xb9q8qiLsFr0=vr0=vr0dc8meaabaqaciaacaGaaeqabaqabeGadaaakeaacqWGubavcqGH9aqpdaaeWbqaamaalaaabaGaemiDaq3aa0baaSqaaiabdQgaQbqaaiabdggaHbaakiabgUcaRiabdogaJnaaDaaaleaacqWGQbGAaeaacqWG1bqDaaGccqGHsisldaqadaqaaiabdsha0naaDaaaleaacqWGQbGAaeaacqWG1bqDaaGccqGHRaWkcqWGJbWydaqhaaWcbaGaemOAaOgabaGaemyyaegaaaGccaGLOaGaayzkaaaabaWaaOaaaeaacqWG0baDdaqhaaWcbaGaemOAaOgabaGaemyyaegaaOGaey4kaSIaem4yam2aa0baaSqaaiabdQgaQbqaaiabdwha1baakiabgUcaRiabdsha0naaDaaaleaacqWGQbGAaeaacqWG1bqDaaGccqGHRaWkcqWGJbWydaqhaaWcbaGaemOAaOgabaGaemyyaegaaaqabaaaaaqaaiabdQgaQjabg2da9iabigdaXaqaaiabdYgaSbqdcqGHris5aOGaaCzcaiaaxMaadaWadaqaaiabbweafjabbghaXjabc6caUiabigdaXaGaay5waiaaw2faaaaa@66EA@

where tja
 MathType@MTEF@5@5@+=feaafiart1ev1aaatCvAUfKttLearuWrP9MDH5MBPbIqV92AaeXatLxBI9gBaebbnrfifHhDYfgasaacH8akY=wiFfYdH8Gipec8Eeeu0xXdbba9frFj0=OqFfea0dXdd9vqai=hGuQ8kuc9pgc9s8qqaq=dirpe0xb9q8qiLsFr0=vr0=vr0dc8meaabaqaciaacaGaaeqabaqabeGadaaakeaacqWG0baDdaqhaaWcbaGaemOAaOgabaGaemyyaegaaaaa@30F2@ and tju
 MathType@MTEF@5@5@+=feaafiart1ev1aaatCvAUfKttLearuWrP9MDH5MBPbIqV92AaeXatLxBI9gBaebbnrfifHhDYfgasaacH8akY=wiFfYdH8Gipec8Eeeu0xXdbba9frFj0=OqFfea0dXdd9vqai=hGuQ8kuc9pgc9s8qqaq=dirpe0xb9q8qiLsFr0=vr0=vr0dc8meaabaqaciaacaGaaeqabaqabeGadaaakeaacqWG0baDdaqhaaWcbaGaemOAaOgabaGaemyDauhaaaaa@311A@ are the number of times allele *j *has been passed on from an heterozygous parent to an affected or unaffected offspring, respectively, cja
 MathType@MTEF@5@5@+=feaafiart1ev1aaatCvAUfKttLearuWrP9MDH5MBPbIqV92AaeXatLxBI9gBaebbnrfifHhDYfgasaacH8akY=wiFfYdH8Gipec8Eeeu0xXdbba9frFj0=OqFfea0dXdd9vqai=hGuQ8kuc9pgc9s8qqaq=dirpe0xb9q8qiLsFr0=vr0=vr0dc8meaabaqaciaacaGaaeqabaqabeGadaaakeaacqWGJbWydaqhaaWcbaGaemOAaOgabaGaemyyaegaaaaa@30D0@ and cju
 MathType@MTEF@5@5@+=feaafiart1ev1aaatCvAUfKttLearuWrP9MDH5MBPbIqV92AaeXatLxBI9gBaebbnrfifHhDYfgasaacH8akY=wiFfYdH8Gipec8Eeeu0xXdbba9frFj0=OqFfea0dXdd9vqai=hGuQ8kuc9pgc9s8qqaq=dirpe0xb9q8qiLsFr0=vr0=vr0dc8meaabaqaciaacaGaaeqabaqabeGadaaakeaacqWGJbWydaqhaaWcbaGaemOAaOgabaGaemyDauhaaaaa@30F8@ are the number of times allele *j *has not been passed on from an heterozygous parent (carrying allele *j*) to an affected or unaffected offspring, respectively, and *l *is the number of alleles at the locus. The TDT test thus took both affected and non-affected siblings into account. A permutation test was carried out according to Lazzeroni and Lange [18], to correct for multiple loci and multiple alleles per locus. For each iteration of the permutation procedure, a permuted data set was made by sampling, for each parent-offspring pair, a haplotype inherited by the offspring from that parent. The two possible, and equally likely, haplotypes were i) the haplotype inherited in the real data, and ii) the haplotype not inherited in the real data. In cases where inheritance from parents to offspring could not be determined in the real data set, because offspring and both parents were heterozygous and identical, linkage phase in the offspring was randomly assigned. TDT statistics were then calculated as above, using the permuted data set. Following the last of *m *permutations, TDT statistics *t*_*i *_for all loci *i *(from the real data set and all permutations) were converted to p-values using the empirical estimate;

pi(ti)=1m∑k=1maki,     [Eq.2]
 MathType@MTEF@5@5@+=feaafiart1ev1aaatCvAUfKttLearuWrP9MDH5MBPbIqV92AaeXatLxBI9gBaebbnrfifHhDYfgasaacH8akY=wiFfYdH8Gipec8Eeeu0xXdbba9frFj0=OqFfea0dXdd9vqai=hGuQ8kuc9pgc9s8qqaq=dirpe0xb9q8qiLsFr0=vr0=vr0dc8meaabaqaciaacaGaaeqabaqabeGadaaakeaacqWGWbaCdaWgaaWcbaGaemyAaKgabeaakmaabmaabaGaemiDaq3aaSbaaSqaaiabdMgaPbqabaaakiaawIcacaGLPaaacqGH9aqpdaWcaaqaaiabigdaXaqaaiabd2gaTbaadaaeWbqaaiabdggaHnaaBaaaleaacqWGRbWAcqWGPbqAaeqaaOGaeiilaWcaleaacqWGRbWAcqGH9aqpcqaIXaqmaeaacqWGTbqBa0GaeyyeIuoakiaaxMaacaWLjaWaamWaaeaacqqGfbqrcqqGXbqCcqGGUaGlcqaIYaGmaiaawUfacaGLDbaaaaa@4B3F@

where *a*_*ki *_= 1 if *T*_*ki *_≥ *t*_*i *_and *a*_*ki *_= 0 otherwise (meaning that *p*_*i*_(*t*_*i*_) is the rate at which the permuted test statistic was larger than the true test statistic). Finally, adjusted p-values (accounting for multiple loci) for the real data were calculated as:

p˜[pi(ti)]=1m∑k=1mbki,     [Eq.3]
 MathType@MTEF@5@5@+=feaafiart1ev1aaatCvAUfKttLearuWrP9MDH5MBPbIqV92AaeXatLxBI9gBaebbnrfifHhDYfgasaacH8akY=wiFfYdH8Gipec8Eeeu0xXdbba9frFj0=OqFfea0dXdd9vqai=hGuQ8kuc9pgc9s8qqaq=dirpe0xb9q8qiLsFr0=vr0=vr0dc8meaabaqaciaacaGaaeqabaqabeGadaaakeaacuWGWbaCgaacaiabcUfaBjabdchaWnaaBaaaleaacqWGPbqAaeqaaOWaaeWaaeaacqWG0baDdaWgaaWcbaGaemyAaKgabeaaaOGaayjkaiaawMcaaiabc2faDjabg2da9maalaaabaGaeGymaedabaGaemyBa0gaamaaqahabaGaemOyai2aaSbaaSqaaiabdUgaRjabdMgaPbqabaGccqGGSaalaSqaaiabdUgaRjabg2da9iabigdaXaqaaiabd2gaTbqdcqGHris5aOGaaCzcaiaaxMaadaWadaqaaiabbweafjabbghaXjabc6caUiabiodaZaGaay5waiaaw2faaaaa@4F3B@

where *b*_*ki *_= 1 if *p*_*i*_(*t*_*i*_) ≥ *min*_*m*_, with *min*_*m *_being the smallest p-value obtained across loci for permutation *m*. A Visual Basic program was written to perform this analysis, since, to our knowledge, the permutation option and the option of using both affected and unaffected offspring is not available in any publicly available programs.

In addition to the locus-level TDT described above, TDT was also performed on individual alleles, at single loci or haplotypes. The TDT statistic (T) was calculated using equation 1, and nominal significance levels were used, T being distributed approximately as χ^2 ^with one degree of freedom. For haplotype TDT, only those haplotypes falling in the significant marker regions from the TDT or ASP tests were considered. Haplotypes were retrieved from ALLEGRO output.

#### Information content

The information content (Info) supplied by ALLEGRO is displayed in Figure 1 and 2. The information content ranges from 0 to 1 (with 1 reflecting complete knowledge of inheritance) and provides a measure of to what extend the information present in the pedigrees could be extracted from the available genotypes, compared to a fully informative situation. It therefore allows an overview of which chromosomes were sufficiently covered by markers and in which chromosomal regions additional markers might increase the power of the QTL study considerably.

## Abbreviations

ASP, affected sib pair; LD, linkage disequilibrium; NPL, non-parametric linkage score; TDT, transmission/disequilibrium test; NSA, numbers of shared alleles; info, information content; INSL3, Insulin like receptor 3; MIS, Müllerian inhibiting substance; CGRP, Calcitonin gene-related peptide; ESR1, estrogen receptor 1; COL9A1, collagen typeIXα; COL2A1, collagen type II; INSL5, Insulin-like hormone 5; CYP19A1, cytochrome P450 family19A1; MAS, master assisted selection.

## Authors' contributions

EG coordinated the study, organised and carried out molecular genetic work, performed the statistical analysis of QTL mapping and drafted the paper. MM carried out molecular genetic work. HT was involved in the statistical analysis. HS was involved in planning the project, power calculations, supervising the statistical analysis and writing the paper. SL was involved in planning the project and writing the paper. TM performed the TDT statistical analysis and contributed in writing the paper.
